# Supplementary material for: Parent’s Perception of the Types of Support Given to Families with an Infant with Phenylketonuria
Source: Nutrients. 2023 May 16;15(10):2328. doi: 10.3390/nu15102328 (PMC10222195; doi:10.3390/nu15102328)
Supplement: Supplementary file 1 [file nutrients-15-02328-s001.zip › nutrients-2379492-supplementary.pdf]

Parent's perception on support for children with PKU

1. I confirm I am a parent/carer of a child with PKU (aged 12 years or younger) AND the child is a UK resident.

   ☐ I agree ☐ I disagree

2. Please use the table below to select the age range of all the children in the family (also indicate if they have PKU).

Age	0-1y	2-3y	4-6y	7-9y	10-12y	13+y	Has PKU
Child 1							
Child 2							
Child 3							
Child 4							
Child 5							
Child 6							

3. Do you speak English as your first language?

   ☐ Yes ☐ No

4. What is your relationship with the child?

   ☐ Mother ☐ Father ☐ Guardian ☐ Other ________

5. Are you a single parent?

   ☐ Yes ☐ No ☐ N/A

6. Who else takes care of the child? (Please select all that apply)

   ☐ Nursery ☐ Childminder ☐ Grandparent ☐ Older sibling

   ☐ Other relative ☐ Friend/Neighbour ☐ Other ______________________

7. How did you feel after the initial diagnosis of PKU in your baby? (Please select all that apply)

☐ Sad	☐ Anxious	☐ Overwhelmed	☐ Exhausted
☐ Angry	☐ Neutral	☐ Lonely	☐ Confused
☐ Calm	☐ Relaxed	☐ Other ________________________	

7.a.Please use the box below if you would like to provide any additional information on the question above.

8. When your child started treatment for PKU, did they receive:

- ☐ breastfeeds with PKU infant formula
- ☐ standard infant formula with PKU infant formula
- ☐ don't know

8.a. When feeding with breast milk and PKU infant formula, please select the type of support you had.

- ☐ given loan of breastfeeding pump
- ☐ advice on how to use breastfeeding pump
- ☐ national breastfeeding helpline
- ☐ voluntary breastfeeding support groups
- ☐ advice from the midwife
- ☐ N/A as only used standard infant formula with PKU infant formula

9. During the early years, which of the following healthcare professionals supported you with your child? Please rate this support out of 5 (1 = not very helpful and 5 = very helpful)

	1	2	3	4	5
GP					
Hospital Doctor					
PKU Dietitian					
PKU Nurse					
Health Visitor					
Psychologist					

10. How much do you trust the information given by the healthcare professional? Please rate this trust out of 5 (1 = not very helpful and 5 = very helpful).

	1	2	3	4	5
GP					
Hospital Doctor					
PKU Dietitian					
PKU Nurse					
Health Visitor					
Psychologist					

11. Did you receive the following from your nurse? (Please select all that apply)

- ☐ advice on how to take a blood test
- ☐ advice on when to take a blood test
- ☐ no advice received

12. Did you receive any of the following advice from a health professional? (Please select all that apply

- ☐ dental hygiene
- ☐ vitamin supplements
- ☐ immunisations
- ☐ child development (e.g. help with speech or language)
- ☐ received none of the above

13. Did you receive the following dietary advice from the dietitian during the first year after the diagnosis? Please rate this advice out of 5 (1 = not very good and 5 = very good). If you did not receive the advice, select N/A.

	1	2	3	4	5	N/A
breastfeeding advice						
bottle feeding advice						
principles of low protein diet						
function and choices of low protein substitute						
provided samples of low protein foods and substitutes						
introduction of solid foods (weaning)						
menu choices						
feeding problems						

14. How do you feel after getting information and support from your dietitian? (Please select all that apply)

☐ Happy ☐ Satisfied ☐ Neutral ☐ Disappointed

☐ Frustrated ☐ Anxious ☐ Sad ☐ Angry

☐ Don't know ☐ Other____________________________

If you selected other, please specify

15. Where do you get your support from? When thinking about support, think about who listens to your concerns and makes you feel better. Please rate this support out of 5 (1 = not very helpful and 5 =very helpful).

	1	2	3	4	5
family					
friends					
talking to other parents of children with PKU					
NSPKU					
NSPKU conferences					

16. If you would like to add any other types of support that are not listed in the question above, please use the box below

17. Where do you get your information from? Think about where you would go to ask questions about PKU and seek ongoing advice. Please rate this support out of 5 (1 = not very helpful and 5 = very helpful).

	1	2	3	4	5
family					
friends					
talking to other parents of children with PKU					
information leaflets/booklets					
NSPKU website					
NSPKU conferences					
hospital PKU events					
company learning packages (e.g. Nutricia)					

18. If you would like to add any other places or people where you get your information that is not listed in the question above, please use the box below.

19. How much do you trust the advice on PKU given by your family?

- ☐ Trust
- ☐ Somewhat trust
- ☐ Neutral
- ☐ Somewhat don't trust
- ☐ Don't trust

20. Do you use social media for PKU information?

☐ Yes ☐ No

20.a. How helpful are the following social media for providing you with support? Please rate this support out of 5 (1 = not very helpful and 5 = very helpful).

	1	2	3	4	5
Facebook					
Instagram					
Twitter					
YouTube					
Internet forums (e.g. Mumsnet)					

20.b. How helpful are the following for providing you with PKU information? Please rate this support out of 5 (1 = not very helpful and 5 = very helpful).

	1	2	3	4	5
Facebook					
Instagram					
Twitter					
YouTube					
Internet forums (e.g. Mumsnet)					

21. Do you currently use any PKU apps?

☐ Yes ☐ No

21.a. How many apps do you use? ________________________

22. Are you currently in one or more private Facebook groups for PKU?

☐ Yes ☐ No

22.a. Which Facebook groups have you joined?

22.b. How much do you trust the information in the groups?

☐ Trust

☐ Somewhat trust

☐ Neutral

☐ Somewhat don't trust

☐ Don't trust

22.c. Do you feel you rely on the Facebook group for support?

☐ Yes ☐ No ☐ Don't know

22.d. What do you gain from being in a Facebook PKU group?

23. Do you feel healthcare professionals should be more involved in social media groups?

☐ Yes ☐ No ☐ Don't know ☐ Other ________

23.a. please explain your answer

24. Which of these most apply to you when learning about PKU?

☐ I like to have the dietitian there throughout the process for support

☐ I prefer to get some support and information from the dietitian and go and do my own research

☐ I prefer to be independent in my learning and require minimal support from my dietitian/ other

☐ Other

24.a. If you selected other, please specify

25. How would you prefer to receive your information from your dietitian? Please rate the following out of 5 (1 = not very useful and 5 = very useful)

	1	2	3	4	5
written handouts					
short videos					
picture books					
social media					
apps					
1:1 sessions (in-person/telephone/video call)					
group sessions (in-person/telephone/video call)					

26. If you have any further comments you would like to add about the support you have received or would like to receive, please type here

